# A case report of rare pancreatic lymphoma: wide range of diffuse large B-cell lymphoma located in the body and tail of pancreas

**DOI:** 10.3389/fonc.2025.1563729

**Published:** 2025-04-29

**Authors:** Xiang Song, Weiyu Hu, Li Zhang, Tao Mao, Yingli Zhu, Xiaokai Chen, Linlin Ren

**Affiliations:** ^1^ Department of Gastroenterology, the Affiliated Hospital of Qingdao University, Qingdao, Shandong, China; ^2^ Department of Hepatobiliary and Pancreatic Surgery, The Affiliated Hospital of Qingdao University, Qingdao, Shandong, China; ^3^ Department of Pathology, the Affiliated Hospital of Qingdao University, Qingdao, Shandong, China

**Keywords:** pancreatic lymphoma, diffuse large B-cell lymphoma, diagnosis, pancreatic cancer, postoperative chemotherapy

## Abstract

**Background:**

Pancreatic lymphoma is a rare pancreatic malignancy that is challenging to differentiate from diseases such as pancreatic cancer (PC). Although pathological examination of specimens obtained through surgery or endoscopic ultrasound-guided fine needle aspiration (EUS-FNA) can aid in diagnosis, factors such as the occasional need for surgery and the variability in specimen quality from EUS-FNA complicate the diagnostic process. Misdiagnosis of pancreatic lymphoma as PC often leads to unnecessary surgery. In addition, surgical intervention may be necessary as a second-line treatment option for pancreatic lymphoma patients presenting with severe gastrointestinal symptoms. However, an optimal postoperative treatment strategy remains undefined, particularly in cases with extensive invasion, thereby impacting long-term survival. This lack of consensus underscores the need for further research to establish evidence-based therapeutic protocols.

**Case Description:**

We present the case of a 55-year-old patient (abdominal pain for over one month, intensified for two weeks). Imaging studies suggested a hypodense mass in the tail of the pancreas with ill-defined margins extending to the spleen, as well as a hypodense lesion within the spleen. The patient underwent surgical intervention, and postoperative pathological analysis confirmed the diagnosis of diffuse large B-cell lymphoma (DLBCL). Following surgery, the patient was initially treated with a short-term C2PET oral chemotherapy regimen, which was subsequently transitioned to the R-CHOP regimen. This therapeutic approach resulted in a favorable outcome, with the patient achieving a 5-year survival period. As far as we know, this may be the first reported case of pancreatic lymphoma with such a widespread involvement, in which patient underwent surgery and postoperative chemotherapy and obtained a 5-year survival period.

**Literature Review:**

We reviewed previously reported cases of the DLBCL pancreatic lymphoma located in the body and tail of pancreas, and conducted a comparative analysis.

**Purposes and Clinical Relevance:**

Our objectives are twofold: first, to highlight the critical role of preoperative EUS-FNA and positron emission tomography–computed tomography in the diagnostic evaluation for patients with pancreatic mass and suspected lesions in immune-associated organs; and second, to propose evidence-based recommendations for postoperative chemotherapy in cases of DLBCL involving the pancreas.

## Introduction

Lymphoma is classified into Hodgkin lymphoma (HL) and non-Hodgkin lymphoma (NHL). HL typically presents as a nodular lesion confined to the lymphatic system and rarely invades extranodal organs, while NHL frequently does. The head and neck are the most frequently involved extranodal sites in NHL, followed by the gastrointestinal tract (1), accounting for only about 30% of NHL cases (2). Among these, the most frequently involved organ is the stomach, followed by the small intestine, colon and rectum, and finally the pancreas, which accounts for less than 2% (3). Lymphoma involving the pancreas is referred to as pancreatic lymphoma.

Similarly, most space-occupying lesions in the pancreas are PC, especially, pancreatic ductal adenocarcinoma, and pancreatic lymphoma being much less common. Pancreatic lymphoma is rare, and often arises from the invasion of peripancreatic lymph nodes. The most common histological type of pancreatic lymphoma is diffuse large B cell lymphoma (DLBCL). The patient in this report had DLBCL pancreatic lymphoma that not only affected the body and tail of the pancreas and spleen but also involved the stomach and part of the colon. This so extensive spread of pancreatic lymphoma was reported for the first time.

To facilitate the clinical diagnosis of pancreatic lymphoma, a variety of methods have been employed, including exploratory laparotomy for pancreatic and lymph node biopsy, EUS-FNA, bone marrow biopsy and peripheral lymph node biopsy. However, due to the peculiarities of certain cases, distinguishing pancreatic lymphoma from other conditions such as autoimmune pancreatitis(AIP), pancreatic neuroendocrine tumors (PNETs), and especially pancreatic ductal adenocarcinoma, remains challenging (4–6).

In clinical practice, many misdiagnoses occur due to the difficulty in differentiating pancreatic lymphoma from pancreatic cancer. However, the choices of treatment for them are not the same. Early diagnosis of pancreatic lymphoma based on clinical features may suggest the use of chemotherapy and radiotherapy rather than surgery (7–10). It is very important to summarize the clinical features of pancreatic lymphoma to provide clinical evidence for the diagnosis of pancreatic lymphoma.

In addition, some patients opt for surgical intervention due to misdiagnosis or severe gastrointestinal symptoms. However, there is currently no consensus on the optimal postoperative chemotherapy regimen for pancreatic lymphoma, highlighting an area that requires further exploration. For DLBCL pancreatic lymphoma, postoperative physical condition is often compromised, making it a significant challenge to achieve a survival period of 5 years or longer. This underscores the need for tailored therapeutic strategies to improve long-term outcomes.

## Case report

**Figure 1:**
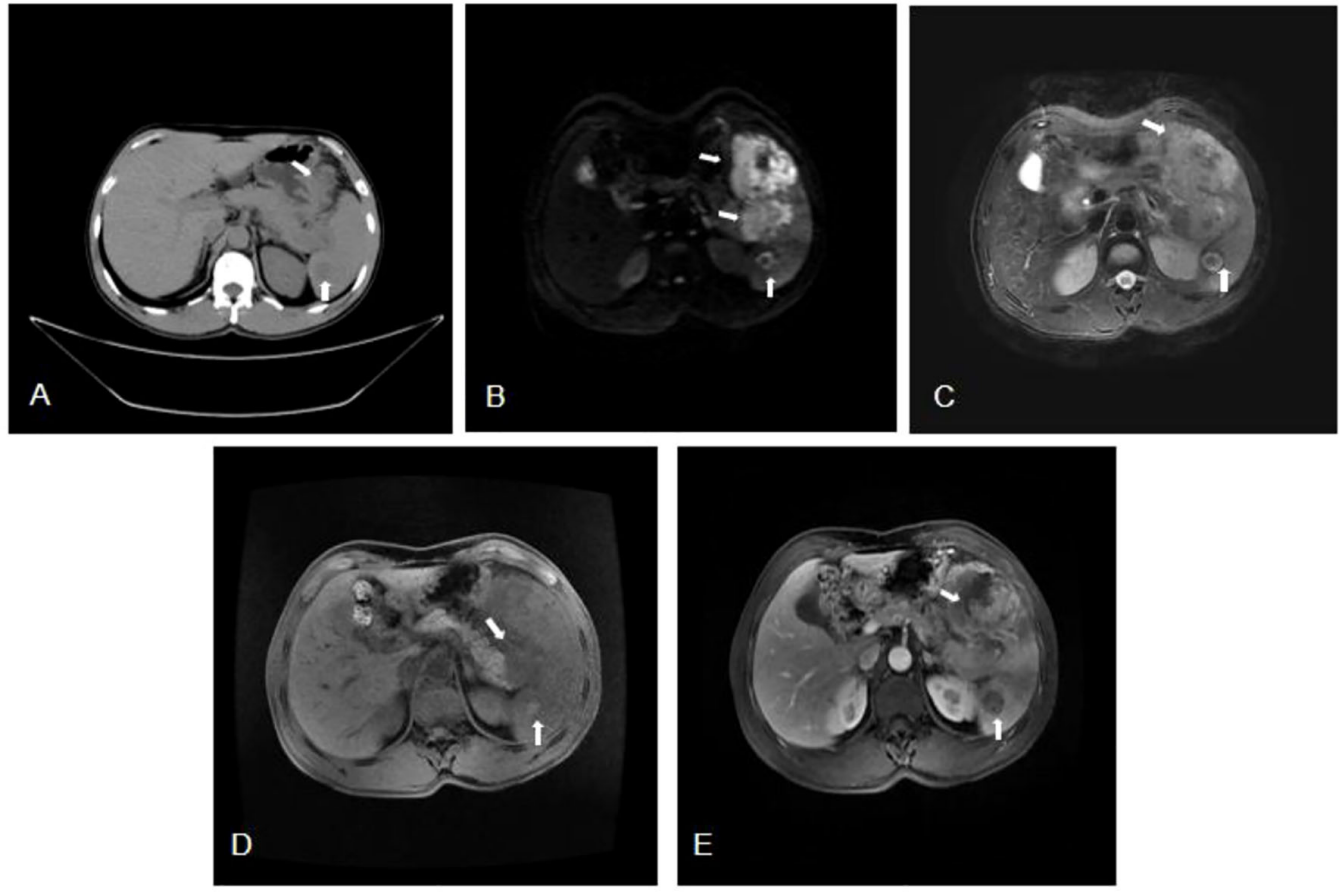
**(A)** Computed tomography (CT) showed a hypodense mass measuring 73mm by 65mm in the tail of the pancreas (arrow), with unclear boundaries extending to the spleen. Additionally, a round-like, slightly hypodense lesion measuring 27mm in diameter (arrow) was observed in the spleen. **(B-D)** Magnetic Resonance Imaging (MRI) showed a soft tissue in the tail of the pancreas, approximately 73mmx65mm in size, exhibiting iso-intensity on T1, mixed T2 signal, and heterogeneous high signal on DWI. The mass had an unclear boundary with the spleen (arrow). And in the spleen, a 27mm round lesion with iso-intensity T1, mixed T2 signal and indistinct circular signal shadow (arrow) was found. **(E)** Magnetic Resonance Enhanced Scan showed a soft tissue mass in the tail of the pancreas and the spleen, which showed irregular enhancement on the contrast-enhanced scan, with a size of about 98mm×95mm (arrow). A long, round T1 signal shadow in the spleen was noted, which did not show significant enhancement, had ill-defined borders, and was approximately 27mm in diameter (arrow).

**Figure 2:**
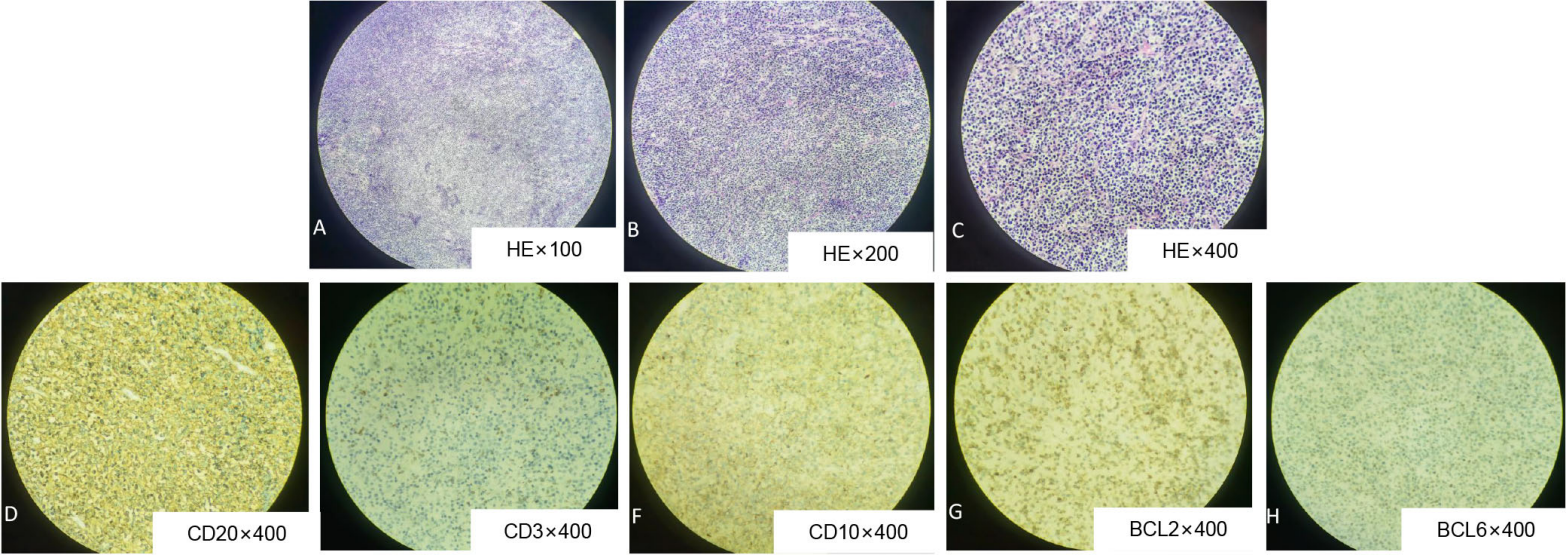
Histological evaluation of pancreatic lesion obtained from DLBCL by surgical resection. Hematoxylin and eosin staining (HE) showed that the atypical lymphocytes of medium size were uniformly and densely distributed (**A**, x100; **B**, x200; **C**, x400). Immunohistochemistry: CD20 **(D)**, CD10 **(F)**, BCL2 **(G)**, BCL6 **(H)** were all positive.

**Figure 3:**
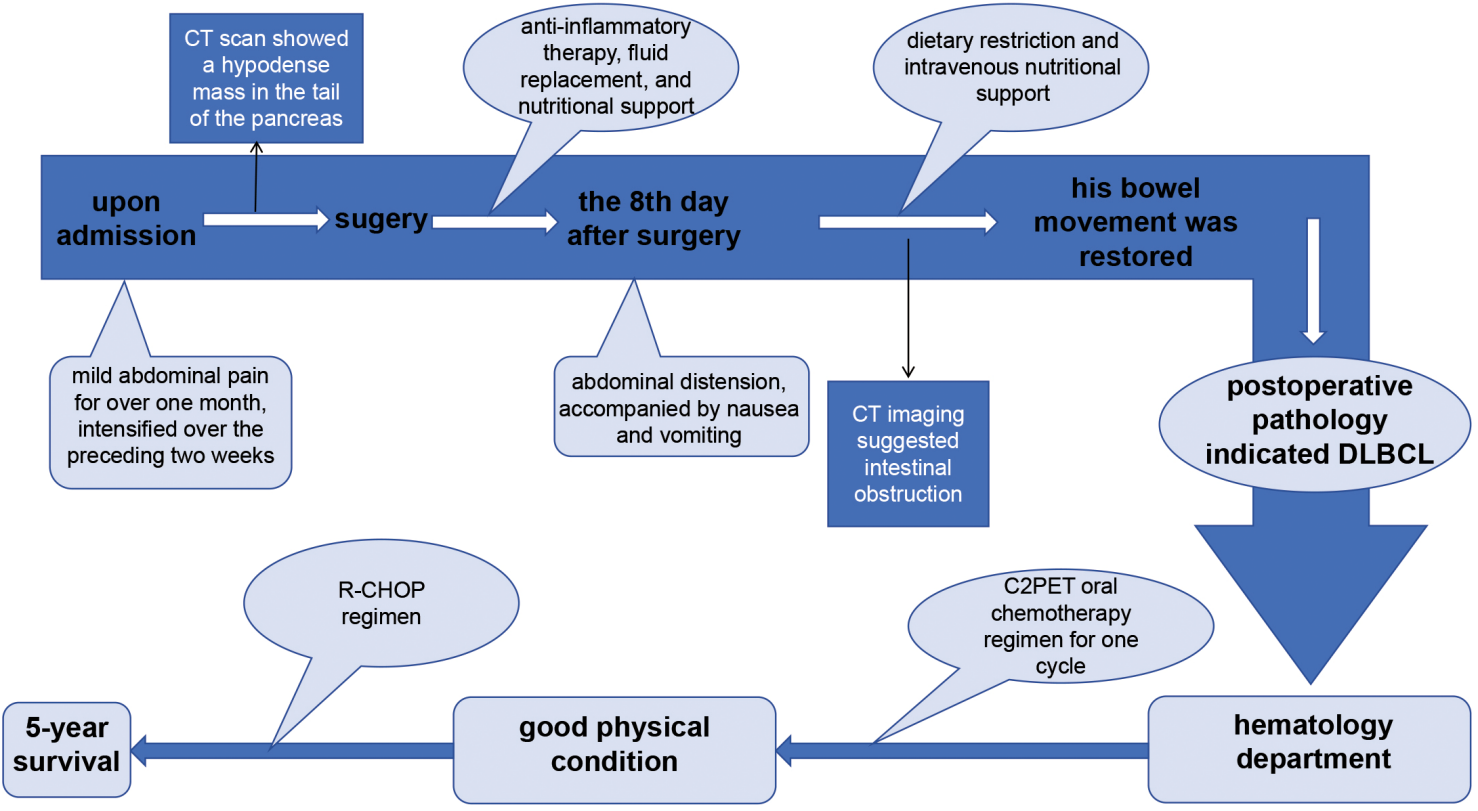
The therapy timeline of the patient reported in this study. The patient was admitted to the hospital with a history of mild abdominal pain persisting for over one month, which had intensified over the preceding two weeks. Imaging studies revealed a hypodense mass in the pancreatic tail, prompting surgical intervention. Postoperative pathological analysis confirmed the diagnosis of diffuse large B-cell lymphoma (DLBCL). Following surgery, the patient was initially treated with a short-term C2PET oral chemotherapy regimen, which was subsequently transitioned to the R-CHOP regimen based on clinical progression. This therapeutic approach resulted in a 5-year survival period in this patient.

The patient, who was a 55-year-old male, was hospitalized with complaints of mild abdominal pain lasting over a month, which worsened in the past two weeks. He did not experience abdominal distention, nausea, vomiting, radiating back pain, diarrhea, jaundice, or pruritus. He suffered from a poor diet and a weight loss of 8kg over one month since the onset of the symptoms. And he had a 20-year history of smoking (1 pack/day) and alcohol consumption (half a kilogram/day). Physical examination revealed no tenderness, jaundice, abdominal mass, peripheral lymph node lesions, or other positive pathological signs. Upper abdominal Computed tomography (CT) scan suggested a hypodense mass measuring 73 mm by 65 mm in the tail of the pancreas, with unclear boundaries extending to the spleen. Additionally, a round-like, slightly hypodense lesion measuring 27mm in diameter was observed in the spleen (**Figure 1A**). Magnetic resonance imaging (MRI) scans showed a mass with soft tissue shadows in the tail of the pancreas, as well as a long T1 and mixed T2 signal round shadow in the spleen (**Figures 1B-D**). Dynamic contrast-enhanced MRI and single organ thin layer scanning revealed a mass in the tail of the pancreas and spleen, with abnormal signal in the spleen which could not exclude hemorrhage, but no evidence of lymph node involvement. Laboratory workup revealed normal CA19-9 (6.92U/ml), CEA (2.18ng/ml), total bilirubin (10.2umol/L), direct bilirubin (3.6umol/L), indirect bilirubin (6.6umol/L), ALP (70.9U/L), total protein (72.1g/L). Prealbumin (154mg/L) was decreased, while LDH (360.6U/L), glycated albumin (15.8%) and CA125 (55.67U/ml) were all elevated. Following the exclusion of surgical contraindications, the patient underwent a comprehensive surgical procedure, which include laparoscopic exploration, open pancreatectomy, splenectomy, colonic splenic flexure resection, partial gastrectomy, and retroperitoneal lymph node dissection. The dissection mass revealed a poorly differentiated malignant tumor in the spleen and tail of the pancreas, measuring 11×7×6cm with nerve invasion. The tumor invaded the pancreatic capsule and splenic capsule, extended to the serosa layer of the stomach and colon, and infiltrated the omentum. A separate nodule in the spleen with a size of 2.4×2.4×2.3cm was founded located 2cm apart from the former lesion, which was also the diagnosed as DLBCL. The pancreatic stump, gastric stump and bilateral intestinal resection margins were negative for tumor involvement, and the pericolonic (0/1), No.7 (0/2), No.8 (0/1) and root of mesentery (0/1) lymph nodes were also not involved. Immunohistochemical analysis revealed positive expression of CD20, CD10, Bcl-2, and BCL6, consistent with the immunophenotypic profile of DLBCL (**Figure 2**). Postoperatively, the patient received anti-inflammatory therapy, fluid replacement, and nutritional support. On postoperative day 8, the patient developed abdominal distension, accompanied by nausea and vomiting, and cessation of bowel movements. Follow-up abdominal CT imaging suggested intestinal obstruction. Management included dietary restriction and intravenous nutritional support, which led to restoration of bowel function. After transitioning to a liquid diet, the patient reported no significant discomforts. The patient was subsequently referred to the hematology department for further management. Bone marrow aspiration and biopsy revealed no abnormal cell population on tumor immunophenotyping, and cytological analysis demonstrated a largely normal bone marrow morphology. Subsequent virological testing detected a significant elevation in cytomegalovirus DNA. The patient declined positron emission tomography-computed tomography (PET-CT) imaging. The patient was diagnosed with high-risk DLBCL, stage IV A, with an age-adjusted International Prognostic Index (aaIPI) score of 2. Due to poor postoperative tolerance, systemic intravenous chemotherapy was deemed unsuitable. Instead, the patient was initiated on the C2PET oral chemotherapy regimen, consisting of cyclophosphamide tablets (100mg daily on days 1-5), prednisone (100mg daily on days 1-5), etoposide (100mg daily on days 1-5) and thalidomide (100mg daily on days 1-14), administered in 21-day cycles. Aspirin (100mg daily at bedtime) was supplemented. After one cycle of the C2PET, the regimen was escalated to R-CHOP: rituximab (600mg on d0), cyclophosphamide (1.2g on d1), pirarubicin (65mg on day1), vindesine (5mg on day 1) and prednisone (100mg daily on days 1-5). The patient survived for 5 years following diagnosis, during which he experienced severe toothache and bowel dysfunction but no other significant complications. The patient’s family expressed satisfaction with the prolonged survival achieved through the treatment regimen. (**Figure 3**).

## Discussion

The case of pancreatic lymphoma reported here was a rare case that occurred in the body and tail of the pancreas and involved a wide range. Typically, pancreatic lymphoma lesions are confined to a localized area of the pancreas or peripancreatic region, however, there have been reports of more extensive cases. For example, one study reported a case of diffuse pancreatic lymphoma affecting the head, body, and tail of the pancreas, with involvement of the spleen and lymph nodes (11). Another report described a case of pancreatic lymphoma with multiple masses distributed in the head, body, and tail of the pancreas (12). In comparison with other reported cases of DLBCL pancreatic lymphoma in the body and tail of the pancreas, the DLBCL pancreatic lymphoma case of our report had a particularly extensive range of involvement. It not only affected the pancreas and spleen, but also extended to part of the stomach and even the colon. To the best of our knowledge, this may be the first report of such extensive involvement, making the clinical, laboratory, imaging findings of this case highly significant.

DLBCL pancreatic lymphoma is more commonly observed in the head, but not the body and tail of pancreas. In contrast, while there have been reports of pancreatic lymphoma occurring in the body of the pancreas (5), such cases are much less common. Due to the unique characteristics of pancreatic lymphoma in this location, its diagnosis, including through CT examination, is more challenging (13). Patients with pancreatic lymphoma can present with varying conditions, clinical manifestations, and examinations results (11, 14–18). A comparison of the patient we reported with other reported cases of DLBCL pancreatic lymphoma occurring in the body and tail of the pancreas is shown in **Table 1**. We observed that the clinical symptoms of lymphoma localized in the body and tail of the pancreas are non-specific, frequently presenting with abdominal pain, while jaundice and abnormal liver function indicators are notably absent. Diagnosing pancreatic lymphoma presents a considerable challenge due to its rarity and its overlapping characteristics with pancreatic adenocarcinoma. Transcutaneous ultrasound, endoscopic ultrasonography, CT and MRI are effective in evaluating pancreatic masses (19, 20). Currently, imaging is the most commonly studied diagnostic tool for differential diagnosis between pancreatic lymphoma and PC (21). CT facilitates a rapid evaluation of tumor size and vascular involvement with high spatial resolution, yet its limited soft tissue contrast may obscure subtle lymphomatous infiltration. On CT, homogeneous low-density masses, as well as masses larger than 6cm, are suggestive of pancreatic lymphoma (11, 22). However, *Arcari* argued that while imaging techniques can raise suspicion of pancreatic lymphoma, it can’t definitively confirm the diagnosis or effectively differentiate pancreatic lymphoma from PC (23). MRI, particularly diffusion-weighted imaging, offers superior soft tissue characterization, enhancing differentiation between lymphoma and necrosis; however, its high cost and prolonged acquisition time constrain its accessibility. Endoscopic ultrasound (EUS) provides exceptional resolution for assessing peripancreatic lymph nodes and enables real-time fine-needle aspiration (FNA) for histopathological confirmation, though its utility is tempered by operator dependence and procedural invasiveness. PET-CT integrates metabolic activity assessment with precise anatomical localization, proving indispensable for staging and treatment monitoring in high-grade lymphomas. However, inflammatory conditions can yield false-positive results, and low-grade subtypes often demonstrate reduced FDG avidity. Biomarkers such as CA19–9 and soluble interleukin-2 receptor (sIL-2R) levels provide non-invasive insights into tumor burden and biological activity, yet their limited specificity necessitates correlation with imaging and histopathology. According to current reports, normal CA19–9 levels, elevated serum sIL-2R levels and a smaller median diameter of the caudal main pancreatic duct in patients with pancreatic space-occupying lesions are strongly indicative of pancreatic lymphoma (6). Based on the cases we reported, we propose that patients presenting with a pancreatic mass accompanied by lesions in immune-associated organs such as the spleen, should be highly suspected of having pancreatic lymphoma. In such cases, further diagnostic evaluation using EUS-FNA combined with PET-CT is recommended. Currently, the ESMO Clinical Practice Guidelines for DLBCL do not provide specific diagnostic recommendations for DLBCL pancreatic lymphoma involving the pancreas, highlighting a critical gap in clinical practice that warrants further attention (24). Therefore, our report is meaningful for summarizing the diagnosis of lymphoma located in the pancreatic body and tail.

**Table 1:** Comparison between the case we reported and DLBCL pancreatic lymphoma occurring in the body and tail of the pancreas in other studies.

Case or reference	Age	Race	Sex	Pancreatic site	Other organs involved	Laboratory Examinations	Main clinical symptoms	Immunohistochemistry	Therapeutic schedule
The case we reported	55	Yellow race	Male	Body and tail of Pancreas	Spleen, gastroomentum, stomach and colon	CA19-9 (6.92U/ml), CEA (2.18ng/ml), total bilirubin (10.2umol/L), direct bilirubin (3.6umol/L), indirect bilirubin (6.6umol/L), ALP (70.9U/L), total protein (72.1g/L) were all normal. Prealbumin (154mg/L) was decreased, while LDH (360.6U/L), glycated albumin (15.8%) and CA125 (55.67U/ml) were all elevated.	Abdominal pain and weight reduction of 8kg	CD20(+), CD10(+), Bcl-2(+), and BCL6(+)	Surgery and postoperative chemotherapy: the short-term C2PET oral chemotherapy regimen, followed by the R-CHOP chemotherapy regimen
(6)	59	White race	Male	Head, body and tail of Pancreas	Spleen and lymphoma		Left sided abdominal pain and early satiety	CD20(+) and LL26(+)	Chemotherapy Protocols: CHOP
(9)	85	White race	Female	Body and tail of Pancreas	No	CA19-9, CA125, LDH andβ2-microglobulin were elevated	Abdominal pain	Dual-expression protein of CMYC and BCL2 proteins	Radiotherapy and chemotherapy were not permitted because of contraindications.
(10)	74	Yellow race	Male	Body and tail of Pancreas	peripancreatic fat	serum amylase and Trypsin were normal, but serum CA19-9 was slightly elevated	Epigastric pain and weight reduction of 5kg		
(11)	41	White race	Female	Body of Pancreas	No	Laboratory Examinations were normal (pregnancy status).	Epigastric pain, with fever, nausea and vomiting		

In addition to pancreatic cancer, pancreatic lymphoma must also be differentiated from other pancreatic diseases through a comprehensive evaluation of clinical, radiological, and histopathological characteristics. In contrast to AIP, which typically presents with diffuse pancreatic enlargement, a characteristic “sausage-shaped” morphology on imaging, and elevated serum IgG4 levels, pancreatic lymphoma more commonly manifests as a localized, bulky mass and lacks IgG4-positive plasma cell infiltration. PNETs frequently exhibit hypervascular enhancement during arterial-phase imaging and may secrete bioactive hormones, leading to distinct clinical syndromes, whereas lymphoma remains hypovascular and hormonally inert. Solid pseudopapillary neoplasms (SPNs), predominantly affecting young females, display a well-circumscribed cystic-solid architecture with internal hemorrhage on imaging. Metastatic pancreatic involvement often presents as multifocal lesions in patients with a known primary malignancy, whereas primary pancreatic lymphoma is typically solitary and associated with regional lymphadenopathy. Histopathologically, lymphoma is characterized by a clonal proliferation of lymphoid cells, distinguishing it from the epithelial-derived SPNs, PNETs, and metastatic tumors.

In terms of treatment, pancreatic lymphoma is primarily treated with chemotherapy, supplemented by radiotherapy and other treatment options. Chemotherapy is generally considered the mainstay of treatment for NHL (25). CHOP, as a standard chemotherapy regimen for many types of lymphoma, is also the most commonly used for pancreatic lymphoma (11). In terms of therapeutic effectiveness, intensive chemotherapy without radiotherapy yields the best outcomes, followed by the CHOP regimen combined with radiotherapy, and finally chemotherapy alone (26, 27). Some studies have raised the question of whether combing intensive chemotherapy with radiotherapy further improves prognosis (28). Due to misdiagnosis or severe gastrointestinal symptoms, some patients with DLBCL involving the pancreas opt for surgical intervention. However, the ESMO Clinical Practice Guidelines for DLBCL do not provide a consensus on the optimal postoperative chemotherapy regimen for such cases, underscoring the need for further exploration in this area (24). For DLBCL patients under 60 years old (aaIPI=2,3) who have not undergone surgery, the ESMO guidelines recommend initiating the R-CHOP regimen directly. However, this may not be well-tolerated by patients recovering from extensive surgical interventions, underscoring a critical gap in current therapeutic recommendations. This discrepancy highlights the need for further investigation into optimized, patient-tailored strategies for postoperative DLBCL management, particularly in surgical compromised populations. In the case we reported, clinicians determined that the patient, who underwent extensive surgery for pancreatic lymphoma with wide-ranging involvement, was in a weakened state and unable to tolerate systemic intravenous chemotherapy. Consequently, the patient was initially treated with a 1-cycle oral C2PET chemotherapy, followed by the R-CHOP regime. This approach resulted a 5-year survival period. Although the patient experienced postoperative bowel dysfunction and end-stage toothache, achieving a 5-year survival in a high-risk DLBCL patient with stage IV disease and an aaIPI score 2 suggests that the chemotherapy regimen employed in this case may hold significant indicative value for similar high-risk patients.

## Conclusion

Currently, the ESMO Clinical Practice Guidelines for DLBCL do not provide specific diagnostic or therapeutic recommendation for DLBCL pancreatic lymphoma occurring in the body and tail of the pancreas (24), highlighting the critical need for further research in this area. Pancreatic lymphoma in the body or tail typically presents with abdominal pain and low back pain as primary symptoms, while rarely manifesting the jaundice or edema commonly associated with pancreatic head cancer, or the classic lymphoma symptoms such as fever and pruritus. Therefore, diagnosis based solely on symptoms is challenging. In addition, imaging and laboratory tests often failed to provide definitive diagnostic clues. However, pancreatic lymphoma should be suspected when CT imaging reveals a mass larger than 6cm with uniform density, particularly when accompanied by normal CA19–9 levels and elevated sIL-2R level. Based on the case we reported, patients presenting with pancreatic mass lesions accompanied by masses in immune-associated organs, even in the absence of lymph node enlargement, should be considered potential pancreatic lymphoma cases. In such instances, a combined diagnostic approach using EUS-FNA and PET-CT is recommended. For DLBCL pancreatic lymphoma patients who undergo surgical intervention due to severe gastrointestinal symptoms or initial misdiagnosis, the therapeutic strategy involving an initial short-term C2PET oral chemotherapy regimen, followed by transition to the R-CHOP chemotherapy regimen tailored to the patient’s condition, appears to be a viable and effective treatment plan. This approach has demonstrated potential in extending patient survival, as evidenced by the favorable outcome in the reported case.

## Data Availability

The original contributions presented in the study are included in the article/supplementary material. Further inquiries can be directed to the corresponding author.
